# Magistral drugs in hospitalized newborns and children

**DOI:** 10.1016/j.rppede.2016.02.012

**Published:** 2016

**Authors:** Agueda Cabral de Souza Pereira, Elaine Silva Miranda, Selma Rodrigues de Castilho, Débora Omena Futuro, Lenise Arneiro Teixeira, Geraldo Renato de Paula

**Affiliations:** Universidade Federal Fluminense (UFF), Niterói, RJ, Brazil

**Keywords:** Medication use, Pediatric medications, Fractionated drugs, Unlicensed drugs

## Abstract

**Objective:**

Study the use of magistral oral solutions and suspensions in infants and children at a university hospital.

**Methods:**

This is a descriptive study based on the analysis of the assessed hospital's magistral drug request forms regarding the patients in the neonatal ICU, Obstetrics, Pediatrics and Pediatric Emergency from January 2012 to December 2013. The frequency of drug requests and dispensation was evaluated and the consumption of each active ingredient of the preparations was expressed as number of “infant defined daily dose” (iDDD) and of iDDD/100 bed-days.

**Results:**

A total of 657 forms were analyzed - a monthly average of 27 pediatric preparations. The neonatal ICU accounted for 69.6% of these requests. Twenty-one drug items were used, of which the most common were folinic acid (88 requests), sulfadiazine (85) and captopril (73). The consumption of the active principle in these preparations varied in number of iDDD, from 7.5 (hydralazine) to 16,520.0 (folic acid), and in number of iDDD/100 bed-days in the neonatal ICU, from 0.1 (zinc sulfate) to 146.1 (folic acid).

**Conclusions:**

The constant consumption of magistral oral solutions and suspensions by newborns and children of the assessed hospital indicates the need for such preparations as a pediatric therapeutic alternative in this hospital.

## Resumo

**Objetivo:**

Estudar o uso de soluções e suspensões orais magistrais em recém-nascidos e crianças de um hospital universitário.

**Métodos:**

Foi feito um estudo descritivo a partir da análise dos formulários de solicitação de manipulação do hospital estudado referentes aos pacientes da UTI-neonatal, obstetrícia, pediatria e emergência pediátrica de janeiro de 2012 a dezembro de 2013. As frequências das solicitações e dispensações desses medicamentos foram avaliadas e o consumo de cada princípio ativo das preparações foram expressos sob a forma de número de *infant defined daily dose* (iDDD) e de iDDD/100 leitos-dia.

**Resultados:**

Foram analisados 657 formulários - média mensal de 27 preparações pediátricas. A UTI-neonatal foi responsável por 69,6% dessas solicitações. Foram usados 21 itens de medicamentos, destacou-se o uso de ácido folínico (88 solicitações), sulfadiazina (85) e captopril (73). O consumo de princípio-ativo nessas preparações variou, em número de iDDD, de 7,5 (hidralazina) a 16.520 (ácido fólico) e em número de iDDD/100 leitos-dia da UTI-neonatal, de 0,1 (sulfato de zinco) a 146,1 (ácido fólico).

**Conclusões:**

O consumo constante das soluções e suspensões orais magistrais pelos recém-nascidos e crianças do hospital estudado indica a necessidade dessas preparações como opção terapêutica pediátrica nesse hospital.

## Introduction

Newborns and children go through physiological changes throughout their development, which interfere with the pharmacokinetics and, consequently, the safety and effectiveness of drug treatment in the pediatric age group. Therefore, studies are needed in each pediatric subpopulation for which their use is intended aiming at safety and efficacy assessment of pediatric drugs.^1^

However, there is a shortage of pediatric drugs in the pharmaceutical industry, which can be explained by economic, ethical and technical issues. This fact makes the magistral preparations advantageous options for obtaining medications with appropriate pharmaceutical form for pediatric use, as they allow dose flexibility and easy drug administration.^1^^,^ 2

In Brazil, the preparation of compounded drugs must comply with the rules of the Collegiate Board Resolution (RDC) 67/2007, which provides for Good Compounding Practices of Magistral and Compounded Drugs for Human Use in pharmacies.^3^ To obtain magistral solutions and oral suspensions, the first choice is the compounding of the active principle; however, these can also be obtained by diluting a liquid formulation (e.g., an injectable dilution), provided it is compatible with oral administration, by pulverizing tablets or removing the powder from the capsule.^4^

This study aims to evaluate the use of magistral oral solutions and suspensions in hospitalized newborns and children.

## Method

A descriptive, retrospective study was carried out based on the analysis of request forms for compounding of oral liquid preparations for newborns and children admitted at Hospital Universitário Antônio Pedro (HUAP) of Universidade Federal Fluminense (UFF), related to the period of January 2012 to December 2013. This is a tertiary and quaternary hospital, has 287 beds and serves the population of the Metropolitan Region II of the State of Rio de Janeiro.

The pediatric oral liquid preparations mentioned in this study were prepared at the pharmacy of the UFF, under the responsibility and guidance of a pharmacist, with formulations being based on scientific literature, with adequate validity and packaging, as well as correct storage recommendations, quality control procedures and medication traceability.

The frequencies of requests for pediatric compounded oral liquid formulations in relation to: the month of request; their active principle; their Anatomical-Therapeutic-Chemical (ATC) classification,^5^ and requesting sectors were calculated.

The administration frequency of magistral oral solutions and suspensions was analyzed for total oral use liquid pharmaceutical forms dispensed by the hospital pharmacy to the neonatal intensive care unit and Pediatrics in 2013.

The annual consumption of infant defined number of daily dose (iDDD), which corresponds to 1/10 of the defined daily dose (DDD), was calculated for each active principle of these preparations, assuming the complete consumption of the compounded product. The number of iDDD/100 bed-days was also calculated for patients in the NICU.^6^ The total occupation of beds in this sector was considered to calculate the number of beds in the NICU.

Descriptive statistics tools such as mean, standard deviation and frequency distributions were used for data analysis.

This study was approved by the Institutional Review Board of the UFF (n. 765.880 de 08/08/2014).

## Results

The study analyzed 657 compounding request forms for oral liquid preparations intended for infants and children in the NICU, obstetrics (rooming-in newborns), pediatrics and pediatric emergency services.

Of the assessed newborn and infant services, the NICU was the unit with the highest number of requests (457; 69.6%), followed by pediatrics (99; 15.1%), obstetrics (71, 10, 8%); pediatric emergency (27; 4.1%) and non-identified sector (3, 0.4%).

Per month, an average of 27 magistral oral solutions and suspensions (standard deviation=9.8) were requested. The months with the highest and lowest number of requests were in March 2013 (45 requests) and December 2012 and November 2013 (both with 10 requests), respectively.

The most often requested drug classes during the study period, according to the ATC, were: C - cardiovascular system (258 requests - especially captopril, spironolactone and furosemide); J - anti-infective for systemic use (91 requests - mainly sulfadiazine) and V - varied (88 requests - corresponding to folinic acid). The least requested class was B - blood and blood-forming organs (13 requests - corresponding to folic acid and sodium citrate).

In 2013, 318 liquid pharmaceutical forms for oral use were dispensed to the NICU and 900 to the Pediatrics unit, of which 209 (65.7%) and 40 (4.4%) were compounded oral solutions and suspensions, respectively.

The frequencies of requests for pediatric compounded oral solutions and suspensions were calculated in relation to the active principle requested for these preparations. The consumption of active principle through the iDDD number of these medications in infants and children was also calculated, as well as the number of iDDD/100 bed-days of patients in the NICU (Table 1).

**Table 1:** Use of non-licensed manipulated oral liquid preparations for newborns and children at a hospital in 2012 and 2013 (n=657).

Magistral oral solution/suspension	Frequency of requests for newborns and children in 2012 and 2013	Established oral iDDD (mg)	N. of iDDD consumed by newborns and children in 2012	N. of iDDD consumed by newborns and children in 2013	N. of iDDD/100 bed-days consumed by the NICU in 2012 and 2013
Folic acid	11	0.04	12,625.0	3,900.0	146.1
Folinic acid	88	6	178.3	237.5	2.6
Ursodeoxycholic acid	5	75	61.3	20.0	0.6
Amiodarone	1	20	48.0	0	0
Caffeine	53	40	212.5	226.3	4.0
Captopril	73	5	2842.0	1652.0	21.8
Sodium citrate	2	^a^	^b^	^b^	^b^
Enalapril	6	1	420.0	660.0	0.0
Spironolactone	67	7.5	1132.8	788.0	9.7
Fluconazole	4	20	75.0	130.0	0
Furosemide	63	4	2558.8	1655.0	11.8
Hydralazine	1	10	7.5	0	0
Hydrochlorothiazide	43	2.5	1976.0	1848.0	33.3
Pyrazinamide	2	150	64.0	0	0
Pyridoxine	1	16	937.5	0	0
Pyrimethamine	63	7.5	528.7	338.0	4.9
Propranolol	4	16	10.3	0	0.1
Ranitidine	18	30	232.5	186.3	3.8
Sildenafil	55	5	621.6	282.0	4.9
Sulfadiazine	85	60	2971.7	3041.7	36.5
Zinc sulfate	12	60	12.3	6.7	0.1

a Either DDD or iDDD was not identified for sodium citrate.

b It was not possible to calculate the number of iDDD consumed by newborns and children and the number of iDDD/100 bed-days consumed by the NICU regarding the oral solutions of sodium citrate due to lack of information on DDD or iDDD for sodium citrate.

## Discussion

The need for the use of magistral preparations to cope with the lack of oral liquid formulations from the pharmaceutical industry was demonstrated in this study, which identified 657 magistral oral solutions and suspensions, regarding 21 different active principles, which have been used in the treatment of newborns and children at HUAP in 2012 and 2013. It is noteworthy that the main characteristics of the assessed hospital are: being a public hospital; providing middle- and high-complexity care; providing specialized outpatient care, urgency, emergency care and hospital admission to patients from the Unified Health System; in addition to teaching (Graduation, Post-Graduation, medical and multiprofessional residency) and research activities, characteristics that are similar to other Brazilian university hospitals.

Other studies also demonstrated the need for these solutions and suspensions for pediatric therapy. Modifications of pharmaceutical drug forms have been reported by Oguz et al. and Khdour et al., who identified, respectively, 20.5% and 5.8% modifications in requests.^7^^,^ 8 Costa et al. found 119 drug adaptations from solid to liquid form and Gavrilov et al. identified two dose modifications in pharmaceutical forms.^9^^,^ 10 Dos Santos and Heineck found that 79 prescription items involved extemporaneous preparations.^11^ Extemporaneous compounded oral preparations are magistral drugs that should be used within 48hours.^3^

Important regulatory initiatives were developed in Europe, United States and Australia to increase the number of available pediatric drugs, but Brazil still lacks specific regulation for the registration and use of pediatric drugs and an incentive policy regarding pediatric research.^9^

Oral solutions and suspensions compounded by the hospital pharmacy or prepared through the modification of the pharmaceutical form performed by the nursing staff are often studied in the category of unlicensed drug use. The definition of unlicensed drug use in pediatrics includes those originated from licensed drugs modifications, such as those that underwent pharmaceutical form modification, those without authorization to be sold from the regulatory agency, imported drugs, the contraindicated drugs and those that have no information on use in this population - although there are divergences among specialists.^12^^,^ 13

The use of unlicensed and off-label medications (of which use is unauthorized by a regulatory agency) in pediatric patients have been the subject of several studies, as they are often prescribed to this population, particularly newborns, despite the high frequency of adverse reactions associated with their use.^13^

Magalhães et al. found that unlicensed drug use in hospitalized pediatric patients occurs in many countries, such as Brazil, Sweden, Croatia, Turkey, Estonia, Netherlands, Palestine, France, Italy, Germany, Finland, Switzerland, Serbia, Spain, Israel and England.^13^ In Brazil, there are few studies on the use of magistral drugs in infants and children, which are concentrated in hospitals from only three Brazilian cities: Fortaleza,^9^^,^ 14 ^,^ 15 Porto Alegre^11^^,^ 16 and Belo Horizonte.^17^^,^ 18

Observing the profile of oral solutions and suspensions compounded for newborns and children of the studied hospital, it was verified that solutions/suspensions of these active principles were identified in other studies: folic acid,^9^^,^ 19 folinic acid,^9^^,^ 20 ursodeoxycholic acid,^11^^,^ 14 captopril,^8^^,^ 9 ^,^ 14 ^,^ 15 ^,^ 18 ^,^ 21 caffeine,^12^^,^ 16 ^,^ 19 ^,^ 22 ^,^ 23 spironolactone,^9^^,^ 24 ^-^ 28 fluconazole,^17^ furosemide,^8^^,^ 9 ^,^ 26 hydrochlorothiazide,^8^^,^ 9 ^,^ 18 ^,^ 26 ^,^ 27 propranolol,^10^ ranitidine,^9^ pyrimethamine^17^ and sulfadiazine.^17^ Therefore, it is observed that the demand for oral solutions and suspensions administered to newborns and children in this study has also been observed in other studies and that the compounding of drugs is a national and international necessity due to the lack of oral liquid drugs from the pharmaceutical industry.

However, it is important to note that some medications that require compounding to be obtained in oral liquid formulation in a certain country may be available in others. In the United States, for instance, the following drugs are approved by FDA and sold as oral solution: caffeine, enalapril, fluconazole, furosemide, propranolol, ranitidine, and sildenafil.^29^

Regarding the recipients of the magistral oral solutions and suspensions, it was observed in the studied hospital that newborns are the main recipients of these formulations and that the NICU is responsible for 70% of these requests. This result is in accordance with that identified by Magalhães et al., which indicate that most unlicensed drugs are prescribed for newborns and often for preterm infants.^13^

The comparison of results on medication use in this study with those of other studies was hindered by methodological differences. Other authors chose to analyze magistral drugs as cases of unlicensed medication use and expressed results as relative frequency (%) of unlicensed items in prescriptions, prescriptions that included unlicensed items and/or patients who received unlicensed drugs. The present study concentrated on oral solutions and suspensions and expressed its results, mainly regarding the amount of active principle consumed in milligrams per number of iDDD and number of iDDD/100 bed-days. The choice of showing the results of this study in relation to iDDD was based on the recommendation made by the WHO on the use of DDD in drug use studies.

It should be observed that the pharmaceutical service division, responsible for adapting active ingredients and/or drugs available on the market for administration in hospitalized patients, is scarce in Brazilian hospitals,^30^ including the studied hospital. Thus, working with the compounding pharmacy is very important to allow the obtaining of magistral drugs necessary for the treatment of newborns and children.

Costa et al. reported that the lack of the drug in appropriate presentation for patient administration led the nursing staff to make dosage form changes when treating patients.^9^^,^ 17 Of the pharmaceutical form adaptations, 75.63% had an inappropriate vehicle and there were also cases of incorrect packaging and conservation.^9^

Among the study limitations are the fact that a single hospital was studied, assuming that each requested preparation was consumed in full and that the NICU occupancy rate was 100% during the studied period. However, it is noteworthy that the studied hospital profile is similar to that of other university hospitals. Additionally, the compounded medication was prepared for a specific patient and had a small volume and narrow expiration date, making it reasonable to assume that the compounded medication was consumed in full. Regarding occupation, the studied hospital is a public reference health unit, which provides care to the population of a densely populated area and the occupancy of 100% of beds is justifiable. Thus, the generalization of the results must take these aspects into account.

It is noteworthy that the use of magistral drugs may be a good therapeutic option for infants and children. However, regulatory incentives are needed for the pharmaceutical industry to produce these drugs at adequate dosage for this population.
